# Who Are They? A Review of Paediatric Presentations to A&E Related to Self-Harm and Suicide

**DOI:** 10.1192/bjo.2025.10177

**Published:** 2025-06-20

**Authors:** Mae Mesgarnezhad, Raffaella Blaylock-Smith, Furhana Ibrahim, Alexandra Battersby

**Affiliations:** 1 Newcastle-upon-Tyne Hospitals, Newcastle-upon-Tyne, United Kingdom; 2 CNTW, Newcastle-upon-Tyne, United Kingdom

## Abstract

**Aims:** Over a six-month period the Great North Children’s Hospital saw more than 150 children presenting with self-harm, suicidal ideation and/or suicide attempts. It is recognised that early identification reduces incidence of self-harm and suicide in children and young people, but how can these patients be identified? The aim of this study was to perform a retrospective review concentrating on *who* attends (sex, age, looked after child status) and *where* patients are presenting from in comparison to areas of deprivation across the region.

**Methods:** A data search performed on all patients attending Paediatric Emergency at the Great North Children’s Hospital with a coding of 'suicidal', 'self-harm', 'intoxicated’ and 'overdose’ returned 271 presentations. Patients were reviewed on a case-by-case basis and removed if deemed to be not meeting criteria (e.g. accidental overdose, no evidence of intent throughout the triage process). Following review, 199 presentations remained of which, taking into account repeat presentations, a total of 158 patients were included in this study. Details for each patient including age, sex, number of presentations in a six-month period, looked after child status, and patient postcodes were recorded.

**Results:** Of the 158 patients assessed the most common presentations were of females aged between 13 and 15 years (58%). 16% of patients were looked after children. Patient postcodes were used to generate a heat map which was then compared with mapping of deprivation across the city of Newcastle-upon-Tyne in the North East of England. This map revealed increased presentations to ED with self-harm, suicidal ideation and suicide attempts amongst patients living in deprived areas.

**Conclusion:** Early identification has been recognised as being effective in preventing self-harm and suicide in children and young people. This study has identified potential key demographics that may be at increased risk: females between the age of 13–15; those who are looked after children; and those from deprived areas. Having identified these at risk-groups further research is warranted to investigate how early help offered to these groups compares with the wider background demographic in order to ascertain whether there is any discrepancy that could be addressed with targeted interventions.

